# Inferring trial-to-trial excitatory and inhibitory synaptic inputs from membrane potential using Gaussian mixture Kalman filtering

**DOI:** 10.3389/fncom.2013.00109

**Published:** 2013-09-03

**Authors:** M. Lankarany, W.-P. Zhu, M. N. S. Swamy, Taro Toyoizumi

**Affiliations:** ^1^Department of Electrical and Computer Engineering, Concordia UniversityMontreal, QC, Canada; ^2^RIKEN Brain Science InstituteSaitama, Japan; ^3^Department of Computational Intelligence and Systems Science, Tokyo Institute of TechnologyYokohama, Japan

**Keywords:** excitatory and inhibitory synaptic inputs, synaptic conductance, Gaussian mixture Kalman filtering, Kalman filtering

## Abstract

Time-varying excitatory and inhibitory synaptic inputs govern activity of neurons and process information in the brain. The importance of trial-to-trial fluctuations of synaptic inputs has recently been investigated in neuroscience. Such fluctuations are ignored in the most conventional techniques because they are removed when trials are averaged during linear regression techniques. Here, we propose a novel recursive algorithm based on Gaussian mixture Kalman filtering (GMKF) for estimating time-varying excitatory and inhibitory synaptic inputs from single trials of noisy membrane potential in current clamp recordings. The KF is followed by an expectation maximization (EM) algorithm to infer the statistical parameters (time-varying mean and variance) of the synaptic inputs in a non-parametric manner. As our proposed algorithm is repeated recursively, the inferred parameters of the mixtures are used to initiate the next iteration. Unlike other recent algorithms, our algorithm does not assume an *a priori* distribution from which the synaptic inputs are generated. Instead, the algorithm recursively estimates such a distribution by fitting a Gaussian mixture model (GMM). The performance of the proposed algorithms is compared to a previously proposed PF-based algorithm (Paninski et al., 2012) with several illustrative examples, assuming that the distribution of synaptic input is unknown. If noise is small, the performance of our algorithms is similar to that of the previous one. However, if noise is large, they can significantly outperform the previous proposal. These promising results suggest that our algorithm is a robust and efficient technique for estimating time varying excitatory and inhibitory synaptic conductances from single trials of membrane potential recordings.

## Introduction

Interaction of the excitatory and inhibitory synaptic inputs constructs the shape of the receptive fields and can elucidate the synaptic mechanism underlying the functional activities of neurons. Therefore, inferring synaptic inputs from neuronal recordings is an important topic of interest in neuroscience (Shu et al., 2003; Wehr and Zador, 2003; Priebe and Ferster, 2005; Murphy and Rieke, 2006; Ozeki et al., 2009; Haider et al., 2013). In many cases, intercellular recordings of membrane potential (or current) under pharmacological blockade spiking activities are used to estimate synaptic inputs. Estimating synaptic inputs based on averaging over many trials and linear regression fitting, which is commonly used, is not always the best methodology because the trial-to-trial variations of synaptic inputs are ignored. The significance of such variations in understanding the neuronal mechanisms of the brain activity (especially spontaneous) and their key roles in information processing is well reviewed in (Destexhe and Contreras, 2006).

The main scope of this paper is to develop an efficient recursive algorithm for estimating synaptic inputs from single trials of recorded membrane potential. We point out two recent studies (Kobayashi et al., 2011; Paninski et al., 2012) that have used the well-known Bayesian approach to infer synaptic inputs in single trials. In both studies, promising results were reported under low observation noise. Kobayashi et al. (2011) considered the Ornstein-Uhlenbeck stochastic model with time-dependent mean and variance as the neuronal model. Kalman filtering (KF) was then used to track these statistical quantities from recorded membrane potential. Paninski et al. (2012) used a compact neuronal model associated with two differential equations representing the dynamics of the excitatory and inhibitory synaptic conductances. Then the sequential Monte-Carlo method [particle filtering (PF)] was derived for filtering/smoothing the dynamics of the model. Finally, an expectation maximization (EM) algorithm (in both parametric and non-parametric manner) was used to infer the time-varying mean of the synaptic conductances. Since the above-mentioned studies used the Bayesian approach, the distributions of synaptic inputs need to be known as *a priori* knowledge. This is the major theoretical drawback of these methods because synaptic distributions are unknown in real neurons. Moreover, Kobayashi et al. (2011) assumed that all excitatory or inhibitory synaptic weights are identical to obtain an explicit relation between the excitatory and inhibitory synaptic inputs vs. the mean and variance of input current, and this assumption does not necessarily hold in real neurons.

The difficulty in estimating the time course of both excitatory and inhibitory synaptic inputs from only a single trial of the recorded data as compared with other conventional methods (averaging and estimating the mean of synaptic inputs) is that the problem is underdetermined since two unknown variables have to be estimated at each time instant. We propose a robust recursive algorithm, based on Gaussian mixture Kalman filtering (GMKF), for filtering/smoothing the dynamics of a compact neuronal model (including synaptic conductances) followed by an EM algorithm to infer the statistical parameters of such synaptic inputs. Our methodology provides more degrees of freedom for these inputs by estimating their distributions with a Gaussian mixture model (GMM). Accordingly, as we are dealing with Gaussian distribution for each mixand, KF is considered as an optimal filtering, which is also faster and easier than the PF approach (Paninski et al., 2012). Once the neuron dynamics are estimated, we can simply (in a closed form) infer the time-varying mean and variance of the synaptic inputs using a non-parametric spline method. Our major contribution in this paper is the development of a general framework for estimating time-varying synaptic conductances when there is no pre-assumption about the synaptic conductances dynamics, e.g., small changes of amplitudes upon presynaptic spikes (Kobayashi et al., 2011), exponential distribution of the synaptic input, or exponential non-linearity to describe the presynaptic input (Paninski et al., 2012). Note that, in the special case of a single Gaussian distribution, our algorithm reduces to the standard KF used in a recursive framework. The simulation results demonstrate the accuracy and robustness of our both KF- and GMKF-based algorithms compared to the PF-based algorithm. While the proposed general GMKF-based algorithm exhibits accurate and robust performance over the entire range of parameters studied, our KF-based algorithm exhibits fast and simple estimation in many representative scenarios. Thus, our general GMKF-based algorithm is a promising tool in neuroscience for estimating excitatory and inhibitory synaptic conductances from single trials of recordings.

The organization of the paper is as follows. In Section Materials and Methods, we introduce the problem of estimating excitatory and inhibitory synaptic conductances (inputs). In addition, sufficient details of our proposed algorithms are explained in this section. In Section Results, we present our simulation results and statistical analysis on the performance of the proposed and existing algorithms. Finally, in Section Discussion, some discussions and concluding remarks are given.

## Materials and methods

### Problem formulation

A reasonable neuronal model similar to Paninski et al. (2012) represents the dynamics of a single-compartment neuron that receives synaptic inputs. The observed membrane potential shows the sub-threshold membrane voltage (active channels are pharmacologically blocked). This model can be expressed as follows.

(1) $$\{\begin{array}{l} V(t+1)=V(t)+dt[g_{L}(E_{L}-V(t))+g_{E}(t)(E_{E}-V(t))\\ \;+\;g_{I}(t)(E_{I}-V(t))]+w(t)\\ g_{E}(t+1)=g_{E}(t)-dt\frac{g_{E}(t)}{\tau_{E}}+N_{E}(t)\\ g_{I}(t+1)=g_{I}(t)-dt\frac{g_{I}(t)}{\tau_{I}}+N_{I}(t) \end{array}$$

where *V*, *g*_*E*_, and *g*_*I*_ are the dynamics of the neuron indicating the membrane potential and excitatory and inhibitory synaptic conductances, respectively, *w*(*t*) is white Gaussian noise of variance σ^2^_*w*_, *N*_*E*_(*t*) and *N*_*I*_(*t*) are the instantaneous excitatory and inhibitory synaptic inputs to the neuron at time step *t* (Koch, 1998; Huys et al., 2006; Paninski et al., 2012), respectively, and *dt* is the time bin that may differ from the voltage recording sampling time (Paninski et al., 2012). Note that the time index *t* takes integer values between 0 and *T*, where *T* × *dt* is the entire (physical) time of recording. We assume that these time steps are equidistant and represent the actual physical sampling duration. Similar to Kobayashi et al. (2011) and Paninski et al. (2012), the reversal potentials *E*_*L*_, *E*_*E*_, and *E*_*I*_, the leakage conductance *g*_*L*_, and the synaptic time constants τ_*E*_ and τ_*I*_ are known. Note that the capacitance of the membrane potential is set to 1 μ F and therefore removed from (1).

Our objective in this paper is to assess the time trace of the excitatory and inhibitory synaptic conductances, *g*_*E*_ and *g*_*I*_, as well as the corresponding synaptic inputs *N*_*E*_ and *N*_*I*_ from noisy membrane potential using the known Bayesian approach. To optimally reconstruct the time course of the excitatory and inhibitory synaptic conductances, we have to determine the probability distributions of the corresponding synaptic inputs, as the *a priori* knowledge in the Bayesian approach. Most of previous studies used Poisson distribution as the distribution of the synaptic inputs (Kobayashi et al., 2011) [see also Paninski et al. (2012) that derived PF for the exponential distribution]. Here we use a weaker assumption about the distributions of the synaptic inputs, namely, the probability distribution function (pdf) of the synaptic input can be estimated by a finite number of weighted Gaussians—GMM. Moreover, by identifying and tracking each Gaussian component with KF, we propose a general GMKF-based algorithm. The pdfs of excitatory and inhibitory synaptic inputs are given by 
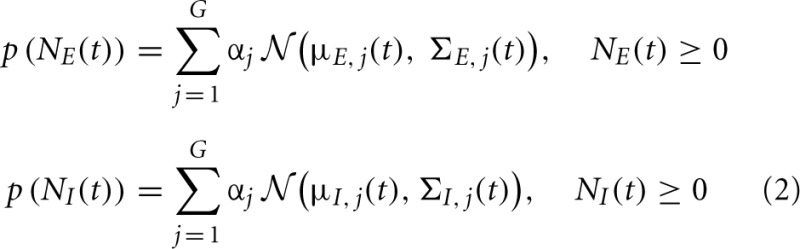
 where μ_*E, j*_(*t*) and μ_*I, j*_(*t*) are, respectively, the mean of the excitatory and inhibitory inputs at time *t* that belong to the *j*^th^ mixand (*j* ∈ {1 : G}). Here, *G* is the number of mixands. Similarly, Σ_*E, j*_(*t*) and Σ_*I, j*_(*t*) are the time-varying variances of these inputs at time *t*, and α_*j*_ is the weight corresponding to selecting the *j*^th^ mixand. Our goal is to estimate *N*_*E*_(*t*) and *N*_*I*_(*t*) in (1) by using the GMM in (2). To this end, we are using extended Kalman filtering (EKF) to estimate the dynamics of (1) followed by the well-known EM algorithm to infer the statistical parameters of the synaptic inputs, μ_*E, j*_(*t*), μ_*I, j*_(*t*), Σ_*E, j*_(*t*), and Σ_*I, j*_(*t*) in (2). By using these statistics as the *a priori* knowledge, we repeat our algorithm until no considerable changes in the estimated dynamics occur.

### Proposed algorithm, Gaussian mixture Kalman filtering

In this subsection, we present GMKF for identifying the excitatory and inhibitory synaptic conductances of a single neuron expressed by (1) from noisy membrane potential. In the following subsections, we use a notation *x*(0:*t*) = {*x*(0), *x*(1), …, *x*(*t*)} to represent the time trace of variable *x* from 0 to *t*. A special case of the GMKF, i.e., KF, will be described later on. Before describing GMKF and KF in detail, we introduce a general recursive framework for estimating (tracking) the parameters (dynamics) of a system whose hidden dynamics are represented by a state space model.

#### General framework

**Figure 1:**
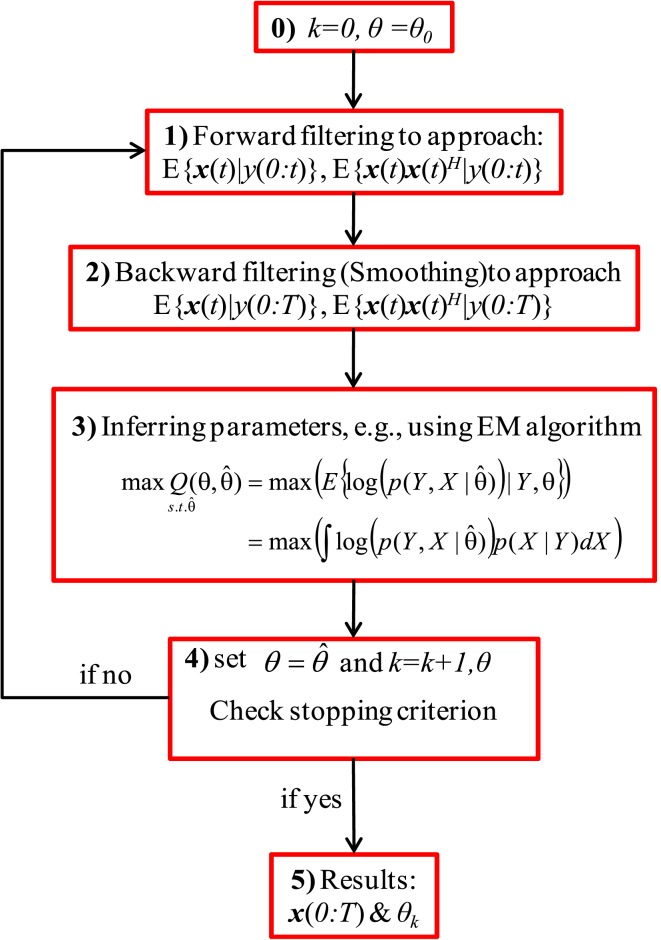
**A block diagram of the general recursive framework**. Schematic representation of the general recursive algorithm is shown for tracking the dynamical states and estimating the time-varying stochastic inputs represented by system (3), where *x* is the state of the system and *y* is the observation. Here, *k* and θ_0_ are the iteration number and the initial values of the statistical parameters, respectively. *X* and *Y* are abbreviations for the entire samples of ***x*** and *y* over time, i.e., *X* = ***x***(0:*T*) and *Y* = *y*(0:*T*). θ is the unknown statistical parameters of the system noise and the super script ^*H*^ represents the matrix transpose operation.

Figure 1 shows a block diagram of the general recursive framework for tracking the hidden dynamics and estimating the (statistical) parameters of a dynamical system, *S*, which is defined as:

(3) $$S:\{\begin{array}{l} x(t+1)=F[x(t)]+v(t)\\ y(t+1)=H[x(t+1)]+\varepsilon (t) \end{array}$$

where ***x***(*t*) and *y*(*t*) indicate, respectively, the state vector and the observation at time *t*, *F* and *H* are the transition and observation functions, and ***v***(*t*) and ε (*t*) are the system noise (or the unknown stochastic inputs) and the observation noise, respectively. In Figure 1, θ stands for the statistical parameters of ***v*** and ε, e.g., the mean and variances. The objective of the recursive algorithm shown in Figure 1 is to estimate/track the dynamics of *S* as well as infer the statistical parameters of the stochastic sources ν and ε. Although this framework has been used in Huys and Paninski (2009) and Paninski et al. (2012), we show the effectiveness and usefulness of this framework for estimating both the hidden states of a system (in a state-space model as well as those modeled as convolution relationship) and the statistics of its input. The recursive algorithm begins with an arbitrary initiation followed by filtering/smoothing steps (2 and 3). These filtering/smoothing steps are necessary to identify the hidden dynamics *S*. Accomplishing this step and calculating the statistics (mean, variance, etc.) of such dynamics, the parameters of the stochastic sources can be inferred by using an appropriate optimization technique, e.g., the EM algorithm. Since these parameters construct the initial values of the next iteration, the algorithm can stop with an appropriate criterion.

#### GMKF-based algorithm

In this subsection, according to aforementioned recursive framework, we derive our algorithm for estimating the excitatory and inhibitory synaptic conductances using GMKF. Let ***x***(*t*) = [*V*(*t*), *g*_*E*_(*t*), *g*_*I*_(*t*)]^*H*^ denotes the vector of neuronal state at time *t*. We can represent the neuronal model (1) by the dynamical system (3) where the observation function is given by *H*[*x*(*t*)] = *C****x***(*t*) with a vector *C* = [1, 0, 0], meaning that only the membrane potential is observed. Similarly, the transition function *F* is given by:

(4) $$\begin{array}{l} F[x(t)]=\left[\begin{array}{ccc} 1-dt\left(g_{L}+g_{E}(t)+g_{I}(t)\right), & dtE_{E}, & dtE_{I}\\ 0, & 1-\frac{dt}{\tau_{E}}, & 0\\ 0, & 0, & \text{1}-\frac{dt}{\tau_{I}} \end{array}\right]\\ \;\left[\begin{array}{l} V(t)\\ g_{E}(t)\\ g_{I}(t) \end{array}\right]+\left[\begin{array}{l} dtg_{L}v_{L}\\ 0\\ 0 \end{array}\right] \end{array}$$

Here, ε is a white Gaussian (observation) noise of variance σ^2^_ε_, and the distribution of the system noise (dynamical noise) ***v***(*t*) *=* [*w*(*t*), *N*_*E*_(*t*), *N*_*I*_(*t*)]^*H*^ [see (1)] is a GMM containing *G* mixands.

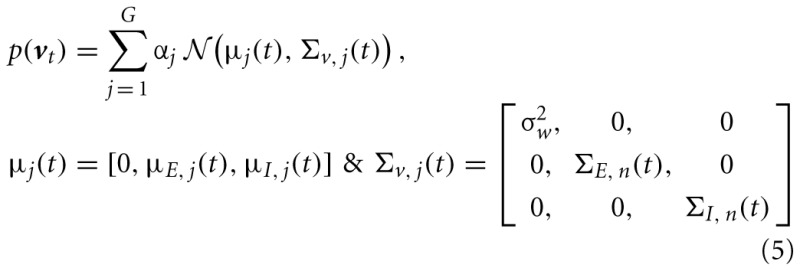



where, α_*j*_ is again the probability of selecting the *j*^th^ mixand, and *N*_*E*_ and *N*_*I*_, which are of our interest, describe excitatory and inhibitory synaptic inputs, respectively. Since the distribution of the system noise is a mixture of Gaussians, one may simply use KF for each mixand. The major drawback of this approach is that the number of Kalman filters required to estimate the conditional probability *p*(***x***(*t*)| *y*(0:*t*)) increases exponentially with time (Kotecha and Djuric, 2003); therefore, computational costs of this approach becomes very heavy. However, to eliminate this drawback, we use a parallel dynamic state space and resampling approach (Jayesh et al., 2003). The aim of this approach is to keep a constant number of Kalman filters for estimating the conditional probability *p*(***x***(*t*)| *y*(0:*t*)) upon arrival of a new observation at *t*. In this regard, the conditional probability *p*(***x***(*t*)|*y*(0:*t*)) is approximated by *K* filters. Then, it is obvious that *K* × *G* Kalman filters are required to represent *p*(***x***(*t*+1)| *y*(0:*t*+1)) (see Appendix 1 for more details). Using a resampling technique, *K* filters are again selected to approximate the later probability; hence, the number of filters remains constant at the arrival of each new observation. Consistent with this description, *p*(***x***(*t*)| *y*(0:*t*)) can be expressed as the combination of *K* parallel Kalman filters, as given below,

(6) $$p(x(t)|y(0:t))=\sum_{i=1}^{K}\beta_{i}(t)p(x(t)|y(0:t),i)$$

where *p*(*x*(*t*)|*y*(0:*t*), *i*) indicates the conditional pdf of the *i*^th^ filter and β_*i*_(*t*) is the normalized weight corresponding to the *i*^th^ Kalman filter at the arrival of a new observation at *t*. At the arrival of a new observation at time instant *t* + 1, the conditional pdf *p*(***x***(*t* + 1)|*y*(0:*t* + 1)) is given by the *K* × *G* parallel Kalman filter [since *p*(*x*(*t* + 1)|*x*(*t*)) is represented by *G* mixands].

(7) $$p(x(t+1)|y(0:t+1))\approx \sum_{i=1}^{K}\sum_{j=1}^{G}\gamma_{i,j}(t+1)p(x(t+1)|y(0:t+1),i,j)$$

where γ_*i, j*_(*t* + 1) is the conditional probability of selecting the *i*th filter and *j*th mixand at the arrival of *y*(*t* + 1), i.e., γ_*i, j*_(*t* + 1) = *p*(*i*, *j*| *y*(0:*t* + 1)). As mentioned above, to avoid increasing the number of filters at each new time, we resample to select the most *K* probable filters from the *K* × *G* filters used in (7). Consequently, (7) can be re-written as

(8) $$p(x(t+1)|y(0:t+1))\approx \sum_{i=1}^{K}\beta_{i}(t+1)p(x(t+1)|y(0:t+1),i)$$

where β_*i*_(*t* + 1) is obtained by selecting the *K* most significant values of γ_*i, j*_(*t* + 1). In the next section, the KF is derived for each *i*∈ {1:K} and *j*∈ {1:G}. The final estimation of the states is the combination of the results of these filters.

***Kalman forward filtering***. In KF, we use a set of mathematical equations underlying the process model to estimate the current state of a system and then correct it using any available sensor measurement (Haykin, 2001). In EKF, the first-order Taylor linearization of the non-linear process and measurement model is used to derive the underlying prediction-correction mechanism. Using (1), *a priori* (predicted) state estimate and error covariance matrix can be calculated at each *t*. Moreover, following the standard KF for linear time invariant systems, the correction step calculates *a posteriori* state estimate and error covariance matrix for this time instant. These variables will be used in the KF recursive framework for the next time instant *t* + 1, regarding the arrival of a new observation. According to the above-mentioned discussions, after combining results from *K* Kalman filters and *G* mixands at *t*, we run *K* × *G* parallel Kalman filters. Then, resampling to select *K* filter is accomplished before the arrival of new observation at *t* + 1. For each *i* belonging to {1:*K*} and *j* belonging to {1:*G*}, we aim to calculate the state estimate *E*{***x***_*i, j*_(*t*)| *y*(0:*t*)} and state correlation matrix *E*{***x***_*i, j*_(*t*)***x***_*i, j*_(*t*)^*H*^| *y*(0:*t*)} in the forward filtering step (see Figure 1) and *E*{***x***_*i, j*_(*t*)| *y*(0:*T*)} and *E*{***x***_*i, j*_(*t*)***x***_*i, j*_(*t*)^*H*^| *y*(0:*T*)} in the backward filtering (smoothing) step using KF approach where *E*{.} stands for the expected value and ***x***_*i, j*_ is the state vector belong to the *i*^th^ filter and *j*^th^Gaussian mixand. For the forward filtering step, for each *i* and *j*, we can apply the EKF approach (Rosti and Gales, 2001) as explained in Appendix 2. After computing *E*{***x***_*i, j*_(*t*)| *y*(0:*t*)} and *E*{***x***_*i, j*_(*t*)***x***_*i, j*_(*t*)^*H*^ | *y*(0:*t*)} in the forward filtering step, resampling is accomplished to select the most probable *K* filters. To do so, γ_*i, j*_(*t*) has to be calculated for each *i*, *j* at *t*. Then, γ_*i, j*_(*t*) can be easily sorted in descending order and the highest *K* values are selected [corresponding to the most probable state estimate *E*{***x***_*i, j*_(*t*)| *y*(0:*t*)} and state correlation matrix *E*{***x***_*i, j*_(*t*)***x***_*i, j*_(*t*)^*H*^| *y*(0:*t*)}].

***Kalman backward filtering (smoothing).*** In this step, we obtain the smoothed state estimate *E*{***x***_*i, j*_(*t*)| *y*(0:*T*)} and state correlation matrix *E*{***x***_*i, j*_(*t*)***x***_*i, j*_(*t*)^*H*^ | *y*(0:*T*)} and the corresponding weights γ_*i, j*_(*t*) for all *i*∈ {1: K}, *j*∈ {1: G}, *t*∈ {1: T}. This step is explained in detail in Appendix 3. Calculating *E*{***x***_*i, j*_(*t*)| *y*(0:*T*)} and *E*{***x***_*i, j*_(*t*)***x***_*i, j*_(*t*)^*H*^ | *y(*0:*T)*} in the backward filtering (smoothing) step, we can infer the statistical parameters of the system noise ***v*** via the EM algorithm.

***Inferring statistical parameters via expectation maximization.*** The EM algorithm is a robust optimization technique for inferring the parameters of models involving unobserved data (Dempster et al., 1977), e.g., the excitatory and inhibitory synaptic inputs *N*_*E*_(*t*) and *N*_*I*_(*t*) in this paper. This algorithm is guaranteed to increase the likelihood of the model at each iteration and therefore can find a local optimum of the likelihood (Paninski et al., 2012). In this section, the EM algorithm is used to infer the statistical parameters of (3–5), i.e., the time varying mean (μ_*j*_(*t*)) and the variance of the states (σ^2^_*w*_, Σ_*v, j*_(*t*)), and the variance of the observation noise (σ^2^_ε_). Having sufficient statistics of the state estimate (mean and correlation matrices) of each mixand *j* and filter *i* (obtained in backward filtering step), we can easily calculate the final state estimate *E*{***x***(*t*)| *y*(0:*T*)} as the combination of the mixtures and parallel filters.

(9) $$E\{x(t)|y(0:T)\}=\hat{x}(t)=\sum_{i}\sum_{j}\gamma_{i,j}(t){\hat{x}}_{i,j}(t)$$

where $\hat{x}(t)={\left[\hat{V}(t),{\hat{g}}_{E}(t),{\hat{g}}_{I}(t)\right]}^{H}$ is the state vector estimated by KF. Note that for the sake of simplicity of expressing notations, we denote *E*{***x***_*i, j*_(*t*)| *y*(0:*T*)} by ${\hat{x}}_{i,\;j}(t)$. To use the EM algorithm, it is essential to write the joint distribution of the states and observation, over time, as follows (*X* and *Y* denote the entire samples of ***x*** and *y* over time, respectively):

(10) $$\begin{array}{l} \;p(Y,X,i,j|\theta )=p(Y|X,\theta ,i,j)p(X|\theta ,i,j)p(i|\theta )p(j|\theta )\\ \log p(Y,X,i,j|\theta )=\log p(i|\theta )+\log p(j|\theta )+\log p(Y|X,\theta ,i,j)\\ \;+\;\log p(X|\theta ,i,j) \end{array}$$

We want to maximize the log of the joint probability of the states and observation via the EM algorithm for each mixture as follows.

(11) $$\begin{array}{l} \underset{s.t.\hat{\theta }}{\max Q}(\theta ,\hat{\theta })=\max\left(E\left\{\log\left(p(Y,X,i,j|\hat{\theta })\right)\text{​}|Y,\theta \right\}\right)\\ \;=\max\left(\int \log\left(p(Y,X,i,j|\hat{\theta })\right)p(X|Y,\theta )dX\right) \end{array}$$

where

(12) $$p(X|Y)=\sum_{i=1}^{K}\sum_{j=1}^{G}p(i,j|Y)p(X|i,j,Y)$$

By doing the corresponding calculations to solve (11) (as described in Appendix 4), we can obtain the mean and variance of each mixand (for both excitatory and inhibitory inputs). By combining them, the total mean and variance of the synaptic inputs as well as the observation noise variance are calculated. As a result, we can update the statistical parameters of the excitatory and inhibitory synaptic inputs as well as the variance of the observation noise in the M-step (see Appendix 4 for full derivation). Inferring all parameters, we can initialize the next iteration of the recursive algorithm. The algorithm continues until no considerable changes in two consecutive iterations occur.

### KF-based algorithm

The simplest case of our GMKF-based algorithm uses a simple Kalman filter (*G* = 1 and *K* = 1) for the filtering/smoothing step. By providing the sufficient statistics in these steps, the non-parametric EM algorithm gives the smoothed mean and variance (both are time-varying) of excitatory and inhibitory synaptic inputs. As a brief description of this algorithm, the pdf *p*(***x***(*t*)| *y*(0:*t*)) is approximated by only one Gaussian distribution. Therefore, *E*{***x***(*t*)| *y*(0:*T*)} [or $\hat{x}(t)$, which is given as a combination of *K* × *G* parallel filters in the GMKF] can be calculated through the standard KF. This strategy not only reduces the complexity of the GMKF-based algorithm but also results in highly accurate reconstruction of the excitatory and inhibitory synaptic conductances in many cases where the true (unknown) distributions of synaptic inputs are nicely approximated by a Gaussian distribution. Otherwise (when the true distributions are far from Gaussian), the estimated parameters from the EM algorithm (which is derived for the Gaussian distribution) give a smoothed version of the underlying true means and variances. Two issues have arisen from the specific choice of *G* = 1 and *K* = 1 that we need to clarify. First, the synaptic conductances have to be constrained as positive values. Second, the EM algorithm has to be derived based on truncated Gaussian distributions for the synaptic inputs. Note that these would not be an issue if *G* > 1 is used because the probability of having negative synaptic conductances naturally decreases with the number of Gaussian mixands. It turns out that neither of these issues affects the estimation of the synaptic conductances in the parameter regime studied here.

The first issue could be easily addressed by using the constrained KF (Gupta and Hauser, 2007), for example, implemented in the convex optimization toolbox CVX (Grant and Boyd, 2012) to penalize the Kalman gain as follows (SDPT3 is another MATLAB package for semi-definite problem optimization that can be used):

(13) $$K^{C}(t)=\arg\min\{trace[(I-K(t)C)\Sigma_{x}^{t-1}(t){\left(I-K(t)C\right)}^{H}+K(t)\sigma_{\varepsilon }^{2}K{\left(t\right)}^{H}]\}$$

(14) $$s.t.\;D(x^{t-1}(t)+K(t)e(t))\geq 0_{3\times 1}$$

where *K*^*C*^(*t*) is the constrained Kalman gain at *t*, *x*^*t* − 1^(*t*) and Σ^*t* − 1^_*x*_(*t*) are the predicted state estimate and state correlation matrix at *t*, respectively, and *D* is diagonal matrix with the values [−1, 1, 1] preserving the negativity of membrane potential (which is not necessary and important for results) as well as the positivity of the synaptic conductances. According to this constraint optimization, the Kalman gain, at each time *t*, is calculated such that the positivity of synaptic conductances is satisfied. It is noteworthy that our results show that a simple constraining on the (updated) state estimate *D****x***^*t*^(*t*) ≧ 0 (***x***^*t*^(*t*) = ***x***^*t* − 1^(*t*) + *K*(*t*)*e*(*t*) in standard KF), without applying the constrained optimization for calculating the new Kalman gain, *K*^*C*^(*t*), has shown very similar performance to that obtained by using (13). This means that the simple and conventional KF with ignoring negative synaptic conductances [zero forcing the updated *x*^*t*^(*t*) for negative synaptic conductances], which we use here, is a reasonable alternative to (13).

The second issue makes the M-step of our EM algorithm more complicated than we have presented for the GMKF-based algorithm. Here again, we have heuristically found that the standard EM algorithm assuming Gaussian distributions of the synaptic conductances works very well because the estimated synaptic conductances rarely take negative values even if the largest noise level explored in this paper is applied.

## Results

### Numerical simulations

We have considered two conditions to analyze the performance of the proposed KF- and GMKF-based algorithms, i.e., the conditions with large and small signal-to-noise ratios (SNRs). First, we conducted two numerical simulations to demonstrate the performance of the KF-based (see Example 1) and GMKF-based (see Example 2) algorithms with large SNR, similar to Paninski et al. (2012), where the variances of system noise (σ^2^_*w*_) and observation noise (σ^2^_ε_) were sufficiently small. Note that estimating excitatory and inhibitory synaptic inputs in this condition was relatively easy and the results did not depend much on the algorithms used. Then, the robustness of the KF- and GMKF-based algorithms were verified in three subsequent examples (see Examples 3–5) with small SNR, in which the PF-based algorithm (Paninski et al., 2012) did not perform well. The time step for our simulations was 2 ms. In all the simulations our recursive algorithm (for both KF- and GMKF-based) ran for 10 iterations, which was consistent with previously used parameters (Paninski et al., 2012) and gave a fair condition to compare our proposed algorithms and the PF-based algorithm. Other model parameters used were similar to Paninski et al. (2012) and summarized in Table 1. We first graphically show the estimation results and later summarize the quantifications in Tables 2, 3.

**Table 1:** **Characteristics of the neuron model**.

*E*_*E*_	10 mV
*E*_*I*_	−75 mV
*E*_*L*_	−60 mV
*g*_*L*_	80 s
τ_*E*_	3 ms
τ_*I*_	10 ms

**Table 2:** **Statistical analysis of the performances of the GMKF-, KF-, and PF-based (Paninski et al., 2012) algorithms in the example with structural synaptic input (specifications of the simulation were the same as in Example 4)**.

**Algorithms\features**	***v***	***g*_*E*_**	***g*_*I*_**
PF	0.0124 ± 1 × 10^−3^	0.5658 ± 5 × 10^−3^	0.3046 ± 3 × 10^−2^
KF	0.0031 ± 0.2 × 10^−3^	0.4106 ± 0.7 × 10^−3^	0.2614 ± 0.5 × 10^−2^
GMKF	0.0033 ± 0.2 × 10^−3^	0.4611 ± 0.7 × 10^−3^	0.2876 ± 0.5 × 10^−2^

The values describe trial means and standard deviations of the normalized estimation error of (15).

**Table 3:** **Statistical analysis of the performances of the GMKF-, KF- and PF-based (Paninski et al., 2012) algorithms in the example with non-structural synaptic input (Specifications of the simulation were the same as in Example 5)**.

**Algorithms\features**	***v***	***g*_*E*_**	***g*_*I*_**
PF	0.0246 ± 7 × 10^−3^	0.6678 ± 0.4 × 10^−2^	0.6479 ± 0.4 × 10^−2^
KF	0.0233 ± 7 × 10^−3^	0.6392 ± 0.6 × 10^−2^	0.6322 ± 0.7 × 10^−2^
GMKF	0.0147 ± 1 × 10^−3^	0.4599 ± 0.6 × 10^−2^	0.5811 ± 0.6 × 10^−2^

Definition of parameters is the same as Table 2.

**Figure 2:**
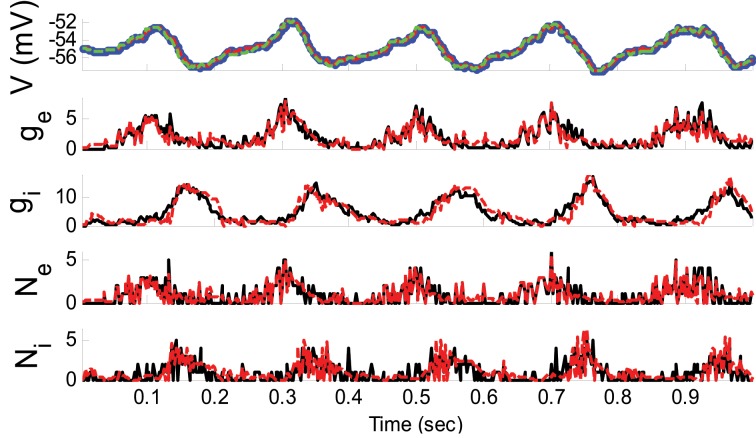
**Estimating synaptic conductances and inputs given a single voltage trace of Example 1 using the KF-based algorithm**. Each panel shows membrane potential (top), excitatory and inhibitory synaptic conductance (second and third from top), and excitatory and inhibitory synaptic inputs (fourth and fifth from top), respectively. Black solid lines represent true values and the red dashed lines represent the estimated ones. The blue dots (in top panel) represent noisy observations of membrane potential. The dashed green line describes the membrane potential reconstructed from the estimated synaptic conductances. The initial values of the KF-based algorithm were set as follows: The time-varying means (for both excitatory and inhibitory) were generated from a uniform distribution and their variances (for both excitatory and inhibitory) were 1 (for all times).

**Example 1**. In this example, the mean pre-synaptic excitatory and inhibitory inputs were a non-linear function of their synaptic fields.

$$\begin{array}{l} E(N_{E}(t))=\exp(\xi_{E}(t))\\ E(N_{I}(t))=\exp(\xi_{I}(t)) \end{array}$$

where ξ_*E*_ and ξ_*I*_ were sinusoidally modulated (5 Hz) input signals (amplitude modulation is 1). ξ_*I*_ had 10 ms delay relative to ξ_*E*_. The synaptic inputs, both excitatory and inhibitory, were generated from a Poisson distribution. The variance of (voltage) system noise (σ^2^_*w*_) was negligible and that of observation noise (σ^2^_ε_) was 0.5 mV. Obviously, since we used a non-parametric EM algorithm, ξ_*E*_ and ξ_*I*_ were unknown. Figure 2 indicates the results of the KF-based algorithm in estimating the excitatory and inhibitory synaptic conductances.

**Figure 3:**
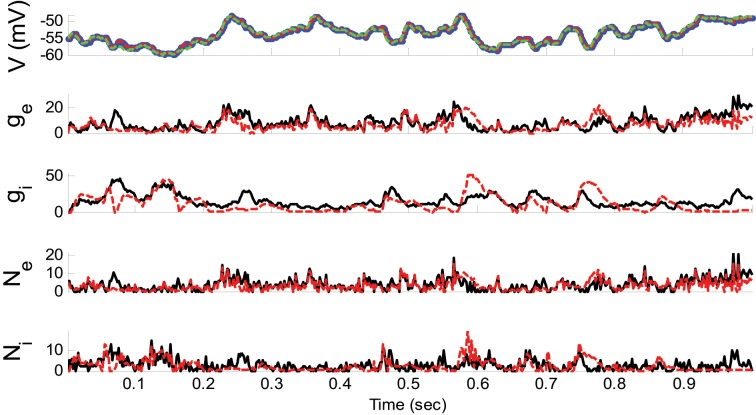
**Estimating excitatory and inhibitory synaptic conductances given a single membrane potential trace of Example 2 using the GMKF-based algorithm**. Other descriptions concerning this figure are the same as those in Figure 2. The initial values of the GMKF-based algorithm (*G* = 2, *K* = 4) were set as follows: the time-varying means (for both excitatory and inhibitory) were generated from a uniform distribution and their variances (for both excitatory and inhibitory) were 1 for both mixands (for all times).

**Example 2**. In this example, the pre-synaptic mean functions were modeled by the absolute value of random realizations of Ornstein-Uhlenbeck processes (a white Gaussian noise is filtered by an exponential filter of amplitude 0.4 and time constant 1.11 ms). The synaptic inputs, both excitatory and inhibitory, were generated from the Poisson distribution and the observation noise was negligible. For the GMKF-based algorithm, we set *G* = 2 (number of mixands) and *K* = 4 (number of Kalman filters; see our discussion about GMKF setting). The variance of the system noise (σ^2^_*w*_) was negligible and that of the observation noise (σ^2^_ε_) was 0.5 mV. Figure 3 shows the results of the GMKF-based algorithm for this example.

**Figure 4:**
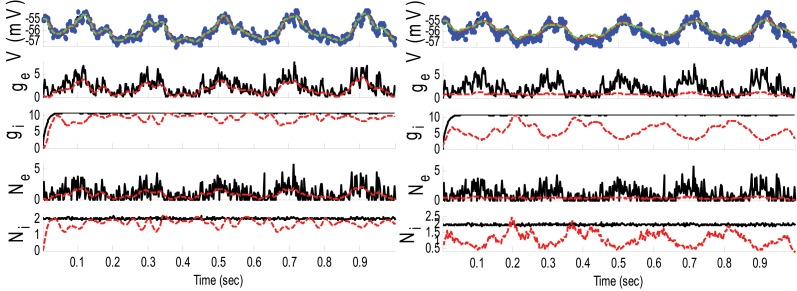
**Estimating synaptic conductances and inputs given a single voltage trace of Example 3 using the KF-based (left) and PF-based (right) algorithms**. Other descriptions about the figure are the same as those in the Figure 2. The initial values of the KF-based algorithm were as follows: the time-varying means (for both excitatory and inhibitory) were generated from a uniform distribution and their variances (for both excitatory and inhibitory) were 5 (for all times). This initial setting (increasing the variance) helped the KF-based algorithm to better estimate the distributions of the synaptic inputs in this example.

**Example 3**. In this example, the mean pre-synaptic input of excitatory was a cosine function (amplitude 1 and frequency of 5 Hz) and that for the inhibitory was a constant value (time-independent). Then, the synaptic inputs were generated from a Gaussian distribution of variance 1.5 and 0.05, respectively, for excitatory and inhibitory inputs. The small variance of the inhibitory synaptic input generated a very narrow distribution function (almost delta function). The variances of the membrane voltage (σ^2^_*w*_) and observation noise (σ^2^_ε_) were 10^−2^ and 5 mV, respectively. These parameters were chosen not because they are physiologically realistic but they illustrate differences in the algorithms. Figure 4 shows the results of the KF- and PF-based algorithms in estimating the synaptic conductances from the observed noisy membrane potential generated in this example.

As can be seen from Figures 2, 3, both the KF- and GMKF-based algorithms accurately identified the excitatory and inhibitory synaptic inputs. These results are not very surprising given the large SNR used in these examples. In fact, the PF-based algorithm could also accurately estimate synaptic inputs under similar conditions (Paninski et al., 2012). In the following examples, we explored cases with a small SNR.

**Figure 5:**
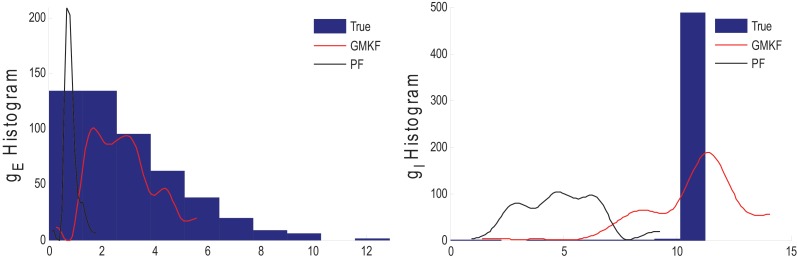
**Histogram of the excitatory (left) and inhibitory (right) synaptic conductances of the true (blue), estimated using the KF-based (red) and the PF-based (black) algorithms in Example 3**.

Figure 4 shows that *g*_*E*_ and *g*_*I*_ as well as the membrane voltage were better estimated using the KF-based algorithm. It is clear that the PF-based algorithm could not track either *g*_*E*_ or *g*_*I*_. Figure 5 shows the distributions of excitatory and inhibitory synaptic conductances. It shows that the KF-based algorithm could estimate the true distributions of *g*_*E*_ and *g*_*I*_ very well, while the PF-based algorithm failed especially for the inhibitory synaptic conductance.

In Example 3, we considered an extreme case in which the inhibitory synaptic input had very narrow distribution. In this case, the KF-based algorithm (by selecting appropriate initiation, i.e., large enough variance) could effectively estimate both excitatory and inhibitory synaptic conductances though the PF-based algorithm completely failed (see Figures 4, 5). Under this small SNR condition, the prior distribution of synaptic input made an important contribution to the results. While the exponential prior distributions assumed for the PF-based algorithm tended to underestimate the inhibitory synaptic input, the KF-based algorithm could better approximate the inhibitory input by fitting a single Gaussian distribution.

**Figure 6:**
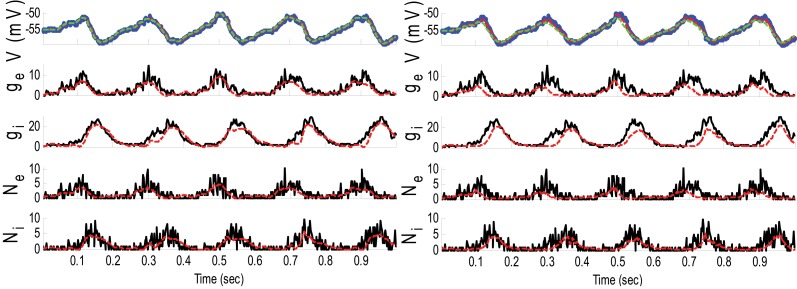
**Estimating synaptic conductances and inputs given a single voltage trace of Example 4 using the GMKF-based (left) and PF-based (right) algorithms**. Other descriptions about the figure are the same as those in Figure 2. The initial values of the GMKF-based algorithm (*G* = 2, *K* = 4) were as follows: the time-varying means (for both excitatory and inhibitory) were generated from a uniform distribution and their variances (for both excitatory and inhibitory) were 0.5 for the first mixand and 2 for the second mixand (for all times).

**Example 4**. In this example, the specifications of the synaptic inputs were the same as those in Example 1 (amplitude modulation is 1.5 for both excitatory and inhibitory inputs). However, the variances of the membrane voltage (σ^2^_*w*_) and observation noise (σ^2^_ε_) increased to 10^−2^ and 5 mV, respectively. Figure 6 shows the results of the GMKF- and PF-based algorithms in estimating the synaptic conductances from the observed noisy membrane potential generated in this example.

**Figure 7:**
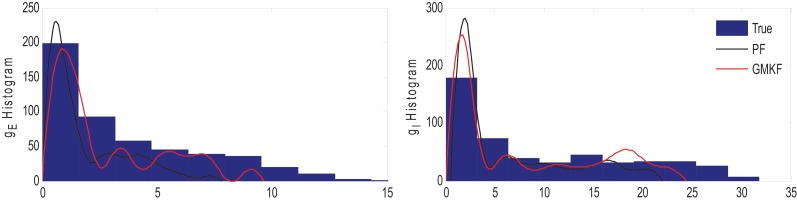
**Histogram of the excitatory (left) and inhibitory (right) synaptic conductance of the true (blue), estimated using the GMKF-based (red) and PF-based (black) algorithms in Example 4**.

The results of each algorithm in Figure 6 confirm that the *g*_*E*_ and *g*_*I*_ (and therefore *N*_*E*_ and *N*_*I*_) were better estimated using the GMKF-based algorithm than the PF-based algorithm. It should be noted that the membrane potential was also better tracked using the GMKF-based algorithm. To see how these algorithms approximate the distributions of the excitatory and inhibitory synaptic conductances, we plotted histograms of *g*_*E*_ and *g*_*I*_ estimated by the GMKF- and PF-based algorithms in Figure 7. As can be seen from Figure 7, the approximated histogram of the GMKF-based algorithm better represents the true distributions for both excitatory and inhibitory synaptic conductances.

**Figure 8:**
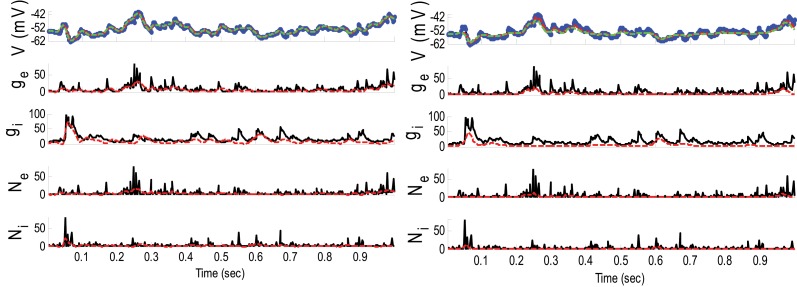
**Estimating synaptic conductances and inputs given a single voltage trace of Example 5 using the GMKF-based (left) and PF-based (right) algorithms**. Other descriptions about the figure were the same as the Figure 2. The initial values of the GMKF-based algorithm (*G* = 2, *K* = 4) were as follows: the time-varying means (for both excitatory and inhibitory) were generated from a uniform distribution and their variances (for both excitatory and inhibitory) were 1 for the first mixand and 4 for the second mixand (for all times).

**Example 5**. In this example, the pre-synaptic mean functions were modeled by the absolute value of random realizations of Ornstein-Uhlenbeck processes (same as Example 2). The synaptic inputs, both excitatory and inhibitory, were generated from the log-normal distribution of variance 1.2. The variances of the membrane voltage (σ^2^_*w*_) and observation noise (σ^2^_ε_) were 10^−2^ and 5 mV, respectively. Figure 8 shows the results of the GMKF-based and PF-based algorithms in estimating the synaptic conductances from the observed noisy membrane potential generated in this example.

**Figure 9:**
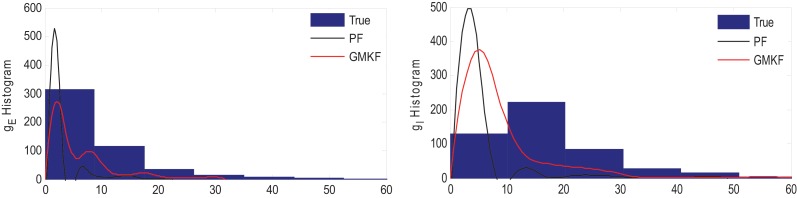
**Histogram of the excitatory (left) and inhibitory (right) synaptic conductances of the true (blue), estimated by GMKF-based (red) and PF-based (black) algorithms in Example 5**.

Similar to Example 4 where the GMKF-based algorithm outperformed the PF-based algorithm, Figure 8 indicates that the *g*_*E*_ and *g*_*I*_ as well as the membrane voltage were better estimated by the GMKF-based algorithm. The estimated *g*_*E*_ and *g*_*I*_ using the PF-based algorithm could not follow the rapid fluctuations of the synaptic conductances. Figure 9 depicts the histogram of the true and estimated *g*_*E*_ and *g*_*I*_ using the GMKF- and PF-based algorithms.

A heavy high-amplitude tail of the distribution of synaptic inputs has often been observed in neuronal circuits (Song et al., 2005; Lefort et al., 2009; Ikegaya et al., 2013). The heavy tail of the log-normal distribution in Example 5 (for both *g*_*E*_ and *g*_*I*_) occasionally produced large synaptic inputs and induced rapid changes in synaptic conductances, which the PF-based algorithm could not keep track of. Hence, this result likely applies to the performance of the GMKF-based vs. PF-based algorithms for heavy-tailed distributions in general. As it is clear from Figures 8, 9, the GMKF-based algorithm could better track synaptic inputs because GMKF (in this example) used two Gaussian mixands that provide more degrees of freedom for fitting the log-normal distribution than only one exponential distribution, which was used in the PF-based algorithm (Paninski et al., 2012).

Theoretically speaking, the PF-based algorithm (Paninski et al., 2012) does not perform accurately under small SNR conditions if the true underlying distributions for synaptic inputs are different from the presumed prior distributions [e.g., an exponential distribution (Paninski et al., 2012)]. Our examples with various distributions of synaptic inputs confirmed that the PF-based algorithm (Paninski et al., 2012) works well if the variance of the observation noise and membrane voltage noise are sufficiently small. The PF-based algorithm can give approximately the same results as the GMKF-based algorithm in this case. However, our examples suggest that the PF-based algorithm does not accurately estimate synaptic inputs from distributions that are not properly approximated by the prior distribution (Examples 3–5) in noisy systems. Under this condition, the GMKF-based algorithm outperforms the PF-based algorithm due to its capability of estimating an arbitrary distribution of synaptic inputs by using a GMM. It should be noted that a larger number of mixands (*G* > 2) may be necessary if the synaptic input distribution is dissimilar to a Gaussian distribution: for example, with a very long tail.

Furthermore, as can be seen from Figures 2–4, 6, 8, the reconstructed membrane potential (dashed green lines) from the estimated synaptic conductances are closely overlap the estimated membrane potential (red dashed line in the top panels) obtained by GMKF and KF (or PF). This suggests that the difference of the estimated membrane potential and the reconstructed membrane potential based on the synaptic conductances was negligible in the range of the noise levels we have examined.

### Statistical analysis

In addition to the above-mentioned observations from the simulation results and in order to compare our algorithms with the PF-based algorithm (Paninski et al., 2012), a statistical analysis was performed in this section. Two types of synaptic inputs, namely, structural (cosine function) and non-structural (O-U process) were considered to generate the membrane potential. Then, each algorithm was applied to 10 trials of these membrane potentials. For the numerical simulations with the structural synaptic input, the same specifications as in Example 4 were used and for the example in which synaptic inputs were generated from the O-U process, the same specifications as in Example 5 were applied. Tables 2, 3 quantify the performance of each algorithm in these examples. For each algorithm, the mean and standard deviation (std) of the normalized error over time was calculated for *V*, *g*_*E*_, and *g*_*I*_ where the normalized error is defined as:

(15) $$err(n)=\frac{\sqrt{\sum_{t=1}^{T}{\left[x_{n}(t)-{\hat{x}}_{n}(t)\right]}^{2}}}{\sqrt{\sum_{t=1}^{T}x_{n}{\left(t\right)}^{2}}}$$

where, *x*_*n*_ and ${\hat{x}}_{n}$ are the true and estimated values of the *n*^th^ trial, respectively. The mean and std were calculated over 10 trials, *err*(*n*)| _*n* = 1:10_.

According to these tables, we can conclude that the performance of our KF- and GMKF-based algorithms was better (for all parameters) than that of the PF-based algorithm. When the synaptic distribution was not heavy-tailed (Table 2), the KF- and GMKF-based algorithms had approximately the same performance. However, for a heavy-tailed synaptic distribution (log-normal in Table 3), the GMKF-based algorithm outperformed the KF-based algorithm. In the GMKF-based algorithm, one could use *G* > 2 (number of mixands) which results in more expensive computations. In our simulations, however, *G* = 2 was sufficiently good to provide the balance between computational costs and accuracy. For very heavy-tailed distributions the higher the value of *G* was the better accuracy was obtained for estimating synaptic inputs. Note that the simulations of (*G* = 2 and *K* = 4, i.e., eight filters for each time) only took approximately the same running time as the PF-based algorithm. Moreover, we observed that *K* = 2, 3, or 4 [number of filters used for estimating *p*(***x***(*t*)| *y*(0:*t*))] did not change the final results considerably. As a rule of thumb, we concluded that, *K* = G is a good choice for selecting the value of *K*. It should be noted that when *G* = 1 and *K* > 1 (the system noise is approximated by only one Gaussian distribution) it is called Gaussian sum filtering [Kalman or particle can be applied, see Kotecha and Djuric, (2003)]. In this case, the conditional probability *p*(***x***(*t*)| *y*(0:*t*)) is estimated using *K* Gaussian filters. However, our case with *G* > 1 and *K* > 1 is called Gaussian mixture filtering. In fact, in this case, *G* > 1 forces *K* to be greater than one in order to better approximate *p*(***x***(*t*)| *y*(0:*t*)), whose filter number grows exponentially overtime [see (Kotecha and Djuric, 2003)]. Note that the number of filters *G* and *K* can be chosen based on a standard model selection criterion such as Akaike's or Bayesian information criterion (Akaike, 1974; Burnham and Anderson, 2002) with real experimental data.

As the main conclusion of our results, we found that our proposed KF- and GMKF-based algorithms outperform the PF-based algorithm. Generically, the GMKF-based algorithm offers a more powerful estimation method than the KF-based algorithm by providing higher degrees of freedom to fit synaptic inputs. However, it is noteworthy that even the KF-based algorithm gives similar results to the GMKF-based algorithm unless the underlying synaptic input distributions are complex. In many cases, the KF-based algorithm is not only much simpler than both the GMKF- and PF-based algorithms but also much faster: therefore, more efficient.

## Discussion

We have proposed a recursive algorithm based on GMKF for estimating the excitatory and inhibitory synaptic conductances (and inputs) from single trials of noisy recorded membrane potential. Two other methods have been proposed in the literature along this direction. Quick alternation of membrane potential between excitatory and inhibitory reversal potentials (Cafaro and Rieke, 2010) enabled nearly simultaneous reconstruction of excitatory and inhibitory synaptic conductances from single trials. One advantage of our method compared to this approach is that it does not require rapid alternations of membrane potential, which might cause experimental artifacts. Thus, our method provides wider applicability to existing as well as future experimental data. Another approach is to infer excitatory and inhibitory synaptic conductances by using the oversampling method (Bédard et al., 2012). Unlike this approach, our KF/GMKF algorithms do not require the manual adjustment of oversampling time steps to suppress singularity problems. The main advantage of our methods in comparison with Kobayashi et al. (2011) and Paninski et al. (2012) relies on the fact that it has the flexibility to estimate an arbitrary (and unknown) pdf of the synaptic inputs by using a GMM. Moreover, we derived and tested a special case of the GMKF-based algorithm when there is only one mixand, i.e., the Kalman filter, for estimating the excitatory and inhibitory synaptic conductances. The simulation results have demonstrated the accuracy and robustness of the proposed algorithms in noisy conditions for estimating synaptic inputs generated from different distributions. In this regard, we have found that the GMKF- and KF-based algorithms outperform the PF-based algorithm (Paninski et al., 2012). We have also found that the GMKF- and KF-based algorithms have approximately identical performances in many cases where simple distributions of synaptic inputs are assumed. On the other hand, the GMKF-based algorithms provide much more accurate estimation than the KF-based one when synaptic inputs are drawn from heavy-tailed distributions with many strong synapses. In practice, running both KF-based and GMKF-based algorithms and comparing their results should provide an idea on how complex the underlying distributions of synaptic inputs are. Therefore, the simplicity and high speed of the KF-based algorithm as well as the robustness and general applicability of the GMKF-based algorithm make them efficient techniques for neuroscientists to monitor trial-to-trial variability of the excitatory and inhibitory synaptic inputs.

### Conflict of interest statement

The authors declare that the research was conducted in the absence of any commercial or financial relationships that could be construed as a potential conflict of interest.

## Appendix 1

In order to show how the number of mixture Kalman filters grows exponentially with the arrival of a new observation *y*(*t*), we assume that *p*(***x***(*t* − 1)| *y*(0:*t* − 1)) can be represented by *K* Kalman filters as follows.

(A1.1) $$p(x(t-1)|y(0:t-1))=\sum_{i=1}^{K}\beta_{i}(t-1)p(x(t-1)|y(0:t-1),i)$$

where β_*i*_(*t* − 1) is the normalized weight corresponding to the *i*^th^ Kalman filter at time *t* − 1. Then, *p*(***x***(*t*)| *y*(0:*t –* 1)) will be given by the following equation considering that *p*(***x***(*t*)| ***x***(*t* − 1)) is approximated by *G* mixands.

(A1.2) $$p(x(t)|y(0:t-1))=\sum_{j=1}^{G}\sum_{i=1}^{K}\alpha_{j}\beta_{i}(t-1)p(x(t)|y(0:t-1),i,j)$$

Now *p*(***x***(*t*)| *y*(0:*t*)) can be calculated using the Bayesian rule.

(A1.3) $$\begin{array}{l} p(x(t)|y(0:t))=\frac{p(y(t)|x(t))p(x(t)|y(0:t-1))}{p(y(t)|y(0:t-1))}\\ \;=\frac{\sum_{j=1}^{G}\sum_{i=1}^{K}\alpha_{j}\beta_{i}(t-1)p(y(t)|x(t),i,j)p(x(t)|y(0:t-1),i,j)}{p(y(t)|y(0:t-1))}\\ \;=\frac{\sum_{j=1}^{G}\sum_{i=1}^{K}\alpha_{j}\beta_{i}(t-1)p(y(t)|y(0:t-1),i,j)p(x(t)|y(0:t),i,j)}{p(y(t)|y(0:t-1))}\\ \;=\sum_{j=1}^{G}\sum_{i=1}^{K}\gamma_{i,j}(t)p(x(t)|y(0:t),i,j) \end{array}$$

where

(A1.4) $$\begin{array}{l} \gamma_{i,j}(t)=p(i,j|y(0:t))\\ \;\propto \frac{\alpha_{j}(t)\beta_{i}(t-1)p(y(t)|y(0:t-1),i,j)}{\sum_{i=1}^{K}\sum_{j=1}^{G}\alpha_{j}(t)\beta_{i}(t-1)p(y(t)|y(0:t-1),i,j)} \end{array}$$

and

(A1.5) $$\begin{array}{l} p(y(t)|y(0:t-1),i,j)=\int p(y(t)|x(t),i,j)p(x(t)|y(0:t-1),i,j)dx(t)\\ \;=\int \int p(y(t)|x(t),i,j)p(x(t)|x(t-1),j)p(x(t-1)|y(0:t-1),i)dx(t)dx(t-1) \end{array}$$

Although (A1.5) can be easily obtained using the results of KF (see Appendix 3), (A1.3) indicates that *p*(***x***(*t*)|*y*(0:*t*)) is approximated by *K* × *G* filters. Consequently, *p*(***x***(*t* + 1)|*y*(0:*t* + 1)) will be represented by *K* × *G*^2^ filters and the number of filters increases exponentially with the arrival of each new observation. As mentioned in Section Results, this problem can be overcome by resampling. To do this, it is required to select the most probable *K* filters of (A1.3) from γ_*i, j*_(*t*). In this regard, *p*(***x***(*t*)|*y*(0:*t*)) will be approximated by only *K* filters as follows.

(A1.6) $$p(x(t)|y(0:t))\approx \sum_{i=1}^{K}\beta_{i}(t)p(x(t)|y(0:t),i)$$

Where β_*i*_(*t*) is the weight corresponding to the *K* highest values of γ_*i, j*_(*t*). Since β_*i*_(*t*) is normalized after the resampling process, it gives equivalent weights to all selected filters (1/*K* for each filter). Please note that the resampling process is done only to eliminate the increase in the number of filters required for approximating *p*(***x***(*t*)|*y*(0:*t*)) at the arrival of new observation *y*(*t*). Hence, *K* × *G* Kalman filters are needed to run at each time *t* to compute *p*(***x***(*t*)|*y*(0:*t*)) for each new observation *y(t)*.

### Appendix 2. Kalman forward filtering (see Rosti and Gales, 2001)

In this appendix, *K* × *G* Kalman forward filtering is derived for each *i* belonging to {1:*K*} and *j* belonging to {1:*G*}. The standard KF technique includes two main steps: 1-time update and 2-measurement update (Haykin, 2001). In the time update step, for each *i* and *j*, the predicted state estimate *E*{***x***_*i, j*_(*t*)| *y*(0:*t* − 1)} and predicted state correlation matrix *E*{***x***_*i, j*_(*t*)***x***_*i, j*_(*t*)^*H*^| *y*(0:*t* − 1)} (therefore the state covariance matrix, *E*{***x***_*i, j*_(*t*)***x***_*i, j*_(*t*)^*H*^| *y*(0:*t* – 1)} − [*E*{***x***_*i, j*_(*t*)| *y*(0:*t* − 1)})^2^] are calculated using (1) [or (3), i.e., the system dynamic]. Then, in the measurement update step, the modified state estimate *E*{***x***_*i, j*_(*t*)| *y*(0:*t*)} and modified state correlation matrix *E*{***x***_*i, j*_(*t*)***x***_*i, j*_(*t*)^*H*^| *y*(0:*t*)} are updated using the recursive Kalman framework. In the following equations, for the sake of simplicity in representing the notations, *E*{***x***_*i, j*_(*t*)| *y*(0:*t* − 1)} and *E*{***x***_*i, j*_(*t*)| *y*(0:*t*)} are denoted as ***x***^*t* − 1^_*i, j*_(*t*) and ***x***^*t*^_*i, j*_(*t*), respectively. Accordingly, the predicted and updated state covariance matrices are denoted as Σ^*t* − 1^_*x, i, j*_(*t*) and Σ^*t*^_*x, i, j*_(*t*), respectively. For each *i* and *j*, the time update step can be followed by

(A2.1) $$\begin{array}{c} \begin{array}{l} \;x_{i,j}^{t-1}(t)=A(t)x_{i}^{t-1}(t-1)+\mu_{v,j}(t-1)\\ \Sigma_{x,i,j}^{t-1}(t)=A(t)\Sigma_{x,i}^{t-1}(t-1)A{\left(t\right)}^{H}+\Sigma_{v,j} \end{array} \end{array}$$

where $A(t)=\frac{\partial F[x]}{\partial }{|}_{x(t)}$ is the time dependent transition matrix, and μ_*v, j*_ and Σ_*v, j*_ are the mean and variance of the synaptic input corresponding to *j*^th^ mixand. The so called Kalman gain is then obtained by

(A2.2) $$K_{i,j}(t)=\Sigma_{x,i,j}^{t-1}(t)C^{H}\Sigma_{y,i,j}^{-1}(t)$$

where

(A2.3) $$\Sigma_{y,i,j}^{\left(t\right)}=C\Sigma_{x,i,j}^{t-1}(t)C^{H}+\sigma_{\varepsilon }^{2}$$

Here, *C* is the observation vector [1, 0, 0] and σ^2^_ε_ is the observation noise variance. By defining the innovation process as, *e*_*i, j*_ (*t*) = *y(t)* − *Cx*^*t − 1*^_*i, j*_(*t*), the updated state estimate and state covariance matrix are calculated as follows.

(A2.4) $$\begin{array}{l} \;x_{i,j}^{t}(t)=x_{i,j}^{t-1}(t)+K_{i,j}(t)e_{i,j}(t)\\ \Sigma_{x,i,j}^{t}(t)=\Sigma_{x,i,j}^{t-1}(t)-K_{i,j}(t)C\Sigma_{x,i,j}^{t-1}(t) \end{array}$$

Since the KF-based algorithm is a recursive algorithm, (A2.4) is used for the next iteration and ***x***^*t*^_*i, j*_(*t*) (or *E*{***x***_*i, j*_(*t*)| *y*(0:*t*)}) is estimated for the all time samples of the observation data *y*(0:*T*).

### Appendix 3. Kalman backward filtering (smoothing) (Rosti and Gales, 2001)

Similar to Appendix 2, Kalman backward filtering is accomplished for *K* × *G* Kalman filters at each iteration, *t*. The resampling procedure is already performed to eliminate the increase in the number of filters estimating the *p*(***x***(*t*)| *y*(0:*t*)). The goal with backward filtering is providing a better estimate of the state mean *E*{***x***_*i, j*_(*t*)| *y*(0:*T*)} and the state correlation matrix *E*{***x***_*i, j*_(*t*)***x***_*i, j*_(*t*)^*H*^| *y*(0:*T*)} using all observed data *y*(0:*T*). Again, for the sake of simplicity, *E*{***x***_*i, j*_(*t*)| *y*(0:*T*)} and the state covariance matrix [*E*{***x***_*i, j*_(*t*)***x***_*i, j*_(*t*)^*H*^| *y*(0:*T*)}—(*E*{***x***_*i, j*_(*t*)| *y*(0:*T*)})^2^] are denoted as ${\hat{x}}_{i,\;j}(t)$ and ${\hat{\Sigma }}_{x,\;i,\;j}(t)$, respectively. Following the standard Kalman backward filtering algorithm, for each *i* belonging to {1:*K*} and *j* belonging to {1:*G*}, we have:

(A3.1) $${\hat{x}}_{i,j}^{t-1}=x_{i,j}^{t-1}(t-1)+J_{i,j}(t-1)\left({\hat{x}}_{i,j}(t)-x_{i,j}^{t-1}(t)\right)$$

where

(A3.2) $$J_{i,j}(t-1)=\Sigma_{x,i,j}^{t-1}(t-1)A{\left(t\right)}^{H}{\left(\Sigma_{x,i,j}^{t-1}(t)\right)}^{-1}$$

The state covariance matrix is calculated as follows.

(A3.3) $${\hat{\Sigma }}_{x,i,j}(t-1)=\Sigma_{x,i,j}^{t-1}(t-1)+J_{i,j}(t-1)\left({\hat{\Sigma }}_{x,i,j}(t)-\Sigma_{x,i,j}^{t-1}(t)\right){\left(J_{i,j}(t-1)\right)}^{H}$$

and ${\hat{\Sigma }}_{x,\;i,\;j}^{t\;-\;1}(t)={\hat{\Sigma }}_{x,\;i,\;j}(t){\left(J_{i,\;j}(t-1)\right)}^{H}$.

For time indices, since in backward filtering the initial values start at *t* = *T*, we have

(A3.4) $${\hat{x}}_{i,j}(T)=x_{i,j}^{T}(T),{\hat{\Sigma }}_{x,i,j}^{T}(T)=\Sigma_{x,i,j}^{T}(T),{\hat{R}}_{i,j}(T)={\hat{\Sigma }}_{x,i,j}^{T}(T)+{\hat{x}}_{i,j}(T){\hat{x}}_{i,j}{\left(T\right)}^{H}$$

Moreover, it is necessary to obtain the correlation matrices *E*{***x***_*i, j*_(*t*)***x***_*i, j*_(*t*)^*H*^| *y*(0:*T*)} and *E*{***x***_*i, j*_(*t*)***x***_*i, j*_(*t* − 1)^*H*^| *y*(0:*T*)} for the expectation maximization (EM) algorithm. These matrices are, respectively, denoted as ${\hat{R}}_{i,\;j}^{t\;-\;1}(t)$ and ${\hat{R}}_{i,\;j}(t)$ in our equations.

(A3.5) $$\begin{array}{c} \begin{array}{l} {\hat{R}}_{x,i,j}(t)={\hat{\Sigma }}_{x,i,j}(t)+{\hat{x}}_{i,j}(t){\left({\hat{x}}_{i,j}(t)\right)}^{H}\\ {\hat{R}}_{x}^{t-1}(t)={\hat{\Sigma }}_{x,i,j}^{t-1}(t)+{\hat{x}}_{i,j}(t){\left({\hat{x}}_{i,j}(t-1)\right)}^{H} \end{array} \end{array}$$

Having the sufficient statistics, we can use the EM algorithm to infer the statistical parameters of each mixture.

### Appendix 4. expectation maximization (EM) algorithm for the GMKF-based algorithm

Recalling (10) and (11), we want to maximize the log of the joint probability of the states and observation via the EM algorithm for each mixture. Then the results are combined to yield the final estimation of the states as well as the distributions of the synaptic inputs.

(A4.1) $$\begin{array}{l} \max Q_{s.t.\hat{\theta }}(\theta ,\hat{\theta })=\max\text{​}\left(\int \sum_{i,j}p(i,j|Y)\log\left(p(Y,X,i,j|\hat{\theta })\right)p(X|i,j,Y)dX\right)\\ \;=\max\text{​}\left(E_{p(X/i,j,Y)}\left\{\sum_{i,j}p(i,j|Y)\log\left(p(Y,X,i,j|\hat{\theta })\right)\text{​}|Y,\theta \right\}\right) \end{array}$$

where *X* and *Y* stand for all the states and observation over time, respectively. The expected value of the joint probability in (A4.1) can be expanded as follows.

(A4.2) $$\begin{array}{l} E_{p(X/n,m,Y)}\left\{\sum_{i,j}p(i,j|Y)\log\left(p(Y,X,i,j|\hat{\theta })\right)\text{​}|Y,\theta \right\}\\ \;=\sum_{i}\sum_{j}\sum_{t=1}^{T}p(i,j|y(0:t))E_{p(X/i,j,Y)}\left\{\log\alpha_{j}(t)+\log\beta_{i}(t)+\log p(y(t)|x(t),i,j,\theta )+\log p(x(t)|x(t),i,j,\theta )\right\}\\ \;=\sum_{i}\sum_{j}\sum_{t=1}^{T}p(i,j|y(0:t))E_{p(X/i,j,Y)}\left\{\log\alpha_{j}(t)\right\}+\sum_{i}\sum_{j}\sum_{t=1}^{T}p(i,j|y(0:t))E_{p(X/i,j,Y)}\left\{\log\beta_{i}(t)\right\}\\ \;+\sum_{i}\sum_{j}\sum_{t=1}^{T}p(i,j|y(0:t))E_{p(X/i,j,Y)}\left\{-\frac{1}{2}\log\sigma_{\varepsilon }^{2}+{\left(y(t)-x(t)\right)}^{H}{\left(\sigma_{\varepsilon }^{2}\right)}^{-1}\left(y(t)-x(t)\right)\right\}\\ \;+\sum_{i}\sum_{j}\sum_{t=1}^{T}p(i,j|y(0:t))E_{p(X/i,j,Y)}\\ \;\left\{-\frac{1}{2}\log\Sigma_{v,i,j}+{\left(x(t)-Ax(t)-\mu_{i,j}(t-1)\right)}^{H}\left(\Sigma_{v,i,j}\right){\text{​}}^{-1}\left(x(t)-Ax(t-1)-\mu_{i,j}(t-1)\right)\right\} \end{array}$$

Considering (A4.2) to solve (A4.1), we need to calculate *E*_*p*(*X*/*i, j, Y*_){***x***(*t*)}, *E*_*p*(*X*/*i, j, Y)*_{***x***(*t* − 1)***x***(*t*)^*H*^} and *E*_*p*(*X*/*i, j, Y)*_{***x***(*t*) ***x***(*t*)^*H*^}. These statistics are already calculated in the backward filtering step as indicated by ${\hat{x}}_{i,\;j}(t)$, ${\hat{R}}_{i,\;j}^{t\;-\;1}(t)$, and ${\hat{R}}_{i,\;j}(t)$, respectively. Furthermore, *p*(*i, j*| *y*(0:*t*)) is already defined in (A1.4) and can be simply computed as follows [calculating the double integral in (A1.5)].

(A4.3) $$\begin{array}{l} p(y(t)|y(0:t-1),i,j)=\int p(y(t)|x(t),i,j)p(x(t)|y(0:t-1),i,j)dx(t)\\ \;=\int \int p(y(t)|x(t),i,j)p(x(t)|x(t-1),j)p(x(t-1)|y(0:t-1),i)dx(t)dx(t-1)\\ \;=\int p(y(t)|x(t),i,j)N(x(t);{\hat{x}}_{i,j}^{t-1}(t),{\hat{\Sigma }}_{i,\;j}^{t-1}(t))dx(t)\\ \;=N\left(y^{t};C{\hat{x}}_{i,j}^{t}(t),\sigma_{\varepsilon }^{2}+C{\hat{\Sigma }}_{i,j}(t)C^{H}\right) \end{array}$$

And *p*(*i, j*| *y*(0:*t*)) can be estimated as

(A4.4) $$\begin{array}{l} p(i,j|y(0:t))=\gamma_{i,j}(t)\\ \;\propto \frac{\alpha_{j}(t)\beta_{i}(t-1)p(y(t)|y(0:t-1),i,j)}{\sum_{i=1}^{K}\sum_{j=1}^{G}\alpha_{j}(t)\beta_{i}(t-1)p(y(t)|y(0:t-1),i,j)} \end{array}$$

Note that β_*i*_(*t* − 1) is the normalized weight after resampling and is equal to 1/*K* for all *t*. Now we can derive the M step by taking the derivative of (A4.2) due to the unknown parameters [α, μ_*v*_, Σ_*v*_, σ^2^_ε_]. It should be noted that it is convenient to demonstrate the optimized parameters in the EM algorithm as $\left[\hat{\alpha },{\hat{\mu }}_{v},{\hat{\Sigma }}_{v},{\hat{\sigma }}_{\varepsilon }^{2}\right]$. For α_*j*_, one can write

(A4.5) $$\underset{s.t.\hat{\theta }}{\max Q}(\theta ,\hat{\theta })=\max\text{​}\left(\sum_{i,j}\sum_{t=1}^{T}\gamma_{i,j}(t)E_{p(X/i,j,Y)}\left\{\log\alpha_{j}(t)\right\}\right)$$

and the fact that $\sum_{j}\alpha_{j}=1$. Using Lagrange multiplier we can solve the following optimization.

(A4.6) $$\sum_{i,j}\sum_{t=1}^{T}\gamma_{i,j}^{t}\log\alpha_{j}-\lambda \left(\sum_{j}\alpha_{j}-1\right)$$

which results in solving the following set of equation.

(A4.7) $$\{\begin{array}{l} \sum_{i=1}^{K}\sum_{t=1}^{T}\gamma_{i,j}(t)\frac{1}{{\hat{\alpha }}_{j}}-\lambda =0\\ \sum_{j}{\hat{\alpha }}_{j}-\text{​}1=0 \end{array}\Rightarrow {\hat{\alpha }}_{j}=\frac{1}{T}\sum_{i=1}^{K}\sum_{t=1}^{T}\gamma_{i,\;j}(t)$$

The optimized ${\hat{\mu }}_{i,\;j}$ can be calculated by taking the derivative of (A4.2) due to this parameter, as given below:

(A4.8) $$\begin{array}{cc} \begin{array}{l} \frac{\partial Q(\theta ,\hat{\theta })}{\partial \hat{\mu }}=\frac{\partial \text{​}\left(\sum_{i,j}\sum_{t=1}^{T}\gamma_{i,j}(t)E_{p(X/i,j,Y)}\left\{-\frac{1}{2}\log\Sigma_{v,i,j}+{\left(x(t)-Ax(t-1)-{\hat{\mu }}_{i,j}(t-1)\right)}^{H}{\left(\Sigma_{v,i,j}\right)}^{-1}\left(x(t)-Ax(t-1)-{\hat{\mu }}_{i,j}(t-1)\right)\right\}\right)}{\partial \hat{\mu }}\\ \;={\left(\Sigma_{v,i,j}\right)}^{-1}\sum_{i,j}\sum_{t=1}^{T}\gamma_{i,j}(t)\left({\hat{x}}_{i,j}(t)-A{\hat{x}}_{i,j}(t-1)-{\hat{\mu }}_{i,j}(t-1)\right) \end{array} & \left(\text{A}4.8\right) \end{array}$$

As we want to calculate ${\hat{\mu }}_{i,\;j}$ for each *i*, *j*, we can rewrite (A4.8) as

(A4.9) $$\begin{array}{l} \frac{\partial Q(\theta ,\hat{\theta })}{\partial {\hat{\mu }}_{i,j}}=0\Rightarrow \sum_{t=1}^{T}\gamma_{i,j}(t)\left({\hat{x}}_{i,j}(t)-A(t){\hat{x}}_{i,j}(t-1)-{\hat{\mu }}_{i,j}(t-1)\right)=0\\ \;{\hat{\mu }}_{i,j}=\frac{\sum_{t=2}^{T}\gamma_{i,j}(t)\left({\hat{x}}_{i,j}(t)-A(t){\hat{x}}_{i,j}(t-1)\right)}{\sum_{t=2}^{T}\gamma_{i,j}(t)} \end{array}$$

Obviously, (A4.9) can be obtained for each mixand *j* as follows (by combining *K* filters).

(A4.10) $${\hat{\mu }}_{j}=\sum_{i=1}^{K}{\hat{\mu }}_{i,j}=\frac{\sum_{i=1}^{K}\sum_{t=2}^{T}\gamma_{i,j}(t)\left({\hat{x}}_{i,j}(t)-A(t){\hat{x}}_{i,j}(t-1)\right)}{\sum_{i=1}^{K}\sum_{i=2}^{T}\gamma_{i,j}(t)}$$

In the non-parametric approach we are using the goal is to estimate the time-varying mean (and variance) using a (defined) basis function *X* (spline basis function in this paper). As a result, the time-varying means *E*{*N_E, j_*(*t*)} and *E*{*N*_*I,j*_(*t*)} can be estimated as follows (recall that μ_*j*_(*t*) = [0, *E*{*N*_*E,j*_(*t*)}, *E*{*N*_*I,j*_(*t*)}]).

(A4.11) $$\begin{array}{l} E\left\{N_{E,j}(t)\right\}=\frac{\sum_{i=1}^{K}\gamma_{i,j}(t)\left({\hat{g}}_{E,i,j}(t)-a_{E}{\hat{g}}_{E,i,j}(t-1)\right)}{\sum_{i=1}^{K}\gamma_{i,j}(t)}\\ E\left\{N_{I,j}(t)\right\}=\frac{\sum_{i=1}^{K}\gamma_{i,j}(t)\left({\hat{g}}_{I,i,j}(t)-a_{I}{\hat{g}}_{I,i,j}(t-1)\right)}{\sum_{i=1}^{K}\gamma_{i,j}(t)} \end{array}$$

where *g*_*E*_ and *g*_*I*_ are the second and third component of the state vector ***x***, respectively. To model *E*{*N_E, j_*(*t*)} = *X*(*t*)ω_*E,j*_ and *E*{*N*_*I,j*_(*t*)} = *X*(*t*)ω_*i,j*_, we need to find the weighting vectors, ω_*E,j*_ and ω_*i,j*_, of the basis function *X* as follows:

(A4.12) $$\begin{array}{c} \begin{array}{l} \omega_{E,j}={\left(X^{T}X\right)}^{-1}X^{T}E\{N_{E,j}\}\\ \omega_{I,j}={\left(X^{T}X\right)}^{-1}X^{T}E\{N_{I,j}\} \end{array} \end{array}$$

where *E*{***N***_*E, j*_} and *E*{***N***_*i, j*_} indicate *E*{*N*_*E, j*_(*t*)} and *E*{*N*_*I, j*_(*t*)} over the entire time. Similar to Paninski et al. (2012), the basis function *X* consists of 50 spline basis functions.

Similarly, the covariance matrix ${\hat{\Sigma }}_{v,i,j}$ can be inferred by taking the derivative of (A4.1) due to this parameter.

(A4.13) $$\begin{array}{l} \frac{\partial Q(\theta ,\hat{\theta })}{\partial {\hat{\Sigma }}_{v,i,j}^{-1}}=\frac{\partial \left(\sum_{i,j}\sum_{t=1}^{T}\gamma_{i,j}(t)E_{p(X/i,j,Y)}\left\{-\frac{1}{2}\log\Sigma_{v,i,j}+{\left(x(t)-A(t)x(t-1)-{\hat{\mu }}_{i,j}(t-1)\right)}^{H}{\left({\hat{\Sigma }}_{v,i,j}\right)}^{-1}(x(t)-A(t)x(t-1)-{\hat{\mu }}_{i,j}(t-1))\right\}\right)}{\partial {\hat{\Sigma }}_{v}^{-1}}\\ \;=\frac{1}{2}\sum_{i,j}\sum_{t=1}^{T}\gamma_{i}(t)\text{​}\left(\Sigma_{v,i,j}-\left({\hat{x}}_{i,j}(t)-A(t){\hat{x}}_{i,j}(t-1)-{\hat{\mu }}_{i,j}(t-1)\right){\left({\hat{x}}_{i,j}(t)-A(t){\hat{x}}_{i,j}(t-1)-{\hat{\mu }}_{i,j}(t-1)\right)}^{H}\right) \end{array}$$

Then, for each *i* and *j* we have

(A4.14) $$\begin{array}{l} \frac{\partial Q(\theta ,\hat{\theta })}{\partial {\hat{\Sigma }}_{v,i,j}^{-1}}=\frac{1}{2}\sum_{t=1}^{T}\gamma_{i,j}(t)\left({\hat{\Sigma }}_{v,j}-\left({\hat{x}}_{i,j}(t)-A(t){\hat{x}}_{i,j}(t-1)-{\hat{\mu }}_{i,j}(t-1)\right){\left({\hat{x}}_{i,j}(t)-A(t){\hat{x}}_{i,j}(t-1)-{\hat{\mu }}_{i,j}(t-1)\right)}^{H}\right)=0\\ \;{\hat{\Sigma }}_{v,i,j}=\frac{\sum_{t=2}^{T}\gamma_{i,j}(t)\left({\hat{R}}_{i,j}(t)-\left[A(t)\;{\hat{\mu }}_{i,j}(t-1)\right]\;{\left[{\hat{R}}_{i,j}(t-1)\;{\hat{x}}_{i,j}(t)\right]}^{H}\right)}{\sum_{t=2}^{T}\gamma_{n,m}(t)} \end{array}$$

Similar to (A4.9), (A4.14) can be obtained for each mixand *j* as follows,

(A4.15) $${\hat{\Sigma }}_{v,j}=\frac{\sum_{i=1}^{K}\sum_{t=2}^{T}\gamma_{i,j}(t)\left({\hat{R}}_{i,j}(t)-\left[A(t)\;{\hat{\mu }}_{i,\;j}(t-1)\right]{\left[{\hat{R}}_{i,j}(t-1)\;{\hat{x}}_{i,j}(t)\right]}^{H}\text{​}\right)}{\sum_{i=1}^{K}\sum_{t=2}^{T}\gamma_{i,j}(t)}$$

The time varying variances of the excitatory and inhibitory inputs can be expressed as:

(A4.16) $$\begin{array}{l} {\hat{\Sigma }}_{E,j}(t)=\frac{\sum_{i=1}^{K}\gamma_{i,j}(t)\left({{\hat{R}}_{i,j}(t)|}_{2,2}-\left[a_{E}N_{E,i,j}(t)\right]{\left[{{\hat{R}}_{i,j}(t-1)|}_{2,2}{\hat{g}}_{E,i,j}(t)\right]}^{H}\text{​}\right)}{\sum_{i=1}^{K}\gamma_{i,j}(t)}\\ {\hat{\Sigma }}_{I,j}(t)=\frac{\sum_{i=1}^{K}\gamma_{i,j}(t)\left({{\hat{R}}_{i,j}(t)|}_{3,3}-\left[a_{I}N_{I,i,j}(t)\right]{\left[{{\hat{R}}_{i,j}(t-1)|}_{3,3}{\hat{g}}_{I,i,j}(t)\right]}^{H}\text{​}\right)}{\sum_{i=1}^{K}\gamma_{i,j}(t)} \end{array}$$

where ${{\hat{R}}_{i,\;j}(t)|}_{2,\;2}$ stands for the second row and second column of the matrix ${\hat{R}}_{i,\;j}(t)$. In our non-parametric model, ${\hat{\Sigma }}_{E,\;j}(t)=X(t)\lambda_{E,}{}_{j}$ and ${\hat{\Sigma }}_{I,\;j}(t)=X(t)\lambda_{i,j}$. The weighting vectors λ *_E, j_* and λ_*i,j*_ of basis function *X* can be given by

(A4.17) $$\begin{array}{l} \lambda_{E,j}={\left(X^{T}X\right)}^{-1}X^{T}{\hat{\Sigma }}_{E,j}\\ \lambda_{I,j}={\left(X^{T}X\right)}^{-1}X^{T}{\hat{\Sigma }}_{I,j} \end{array}$$

where ${\hat{\Sigma }}_{E,\;j}$ and ${\hat{\Sigma }}_{I,\;j}$ indicate ${\hat{\Sigma }}_{E,\;j}(t)$ and ${\hat{\Sigma }}_{I,\;j}(t)$ over the entire time, respectively. Hence, the system noise (including synaptic inputs) can be represented by a GMM as follows.

(A4.18) $$\text{v}(t)\approx \sum_{j=1}^{G}{\hat{\alpha }}_{j}N\left({\hat{\mu }}_{j}(t),{\hat{\Sigma }}_{v,j}(t)\right)$$

where ${\hat{\mu }}_{j}(t)$ and ${\hat{\Sigma }}_{v,j}(t)$ are defined by (5) and their components are calculated in this Appendix. Moreover, as mentioned before, we can simply calculate the final state estimate ***x***(*t*) as the combination of *K* × *G* parallel filters at each time *t*.

(A4.19) $$\hat{x}(t)=\sum_{i,j}\gamma_{i,j}(t){\hat{x}}_{i,j}(t)$$

Note that, the variances of the membrane voltage (σ^2^*_w_*) and observation noise (σ^2^_ε_) can be calculated in a straightforward way, as mentioned in Paninski et al. (2012).
